# The effect of sequential perioperative intravenous tranexamic acid in reducing postoperative blood loss and hidden blood loss after posterior lumbar interbody fusion: a randomized controlled trial

**DOI:** 10.3389/fmed.2023.1192971

**Published:** 2023-08-04

**Authors:** Wei Dong, Yi Liang, Dongxu Li, Zhaoxin Ma, Minghuang Cheng, Xiaojun Zhang, Jieliang Shen, Nian Zhou, Jie Hao, Wei Jiang, Zhenming Hu

**Affiliations:** ^1^Department of Orthopedics, The First Affiliated Hospital of Chongqing Medical University, Orthopedic Laboratory of Chongqing Medical University, Chongqing, China; ^2^Department of Neurology, The Second Affiliated Hospital of Chongqing Medical University, Chongqing, China

**Keywords:** spine surgery, posterior lumbar interbody fusion, perioperative blood loss, hidden blood loss, tranexamic acid

## Abstract

**Background:**

Tranexamic acid (TXA) has previously been shown to be effective in reducing intraoperative blood loss (IBL) and transfusion requirements in spine surgery. A conventional TXA regimen is a simple preoperative or intraoperative administration. However, the hyperfibrinolysis caused by surgical trauma lasts at least 24 h, and a single dose of TXA cannot cover the whole process of hyperfibrinolysis. Moreover, its ability to control postoperative blood loss (PBL) may be insufficient. Therefore, this study aimed to explore the effects and safety of sequential perioperative intravenous TXA for reducing bleeding after posterior lumbar interbody fusion (PLIF).

**Methods:**

Patients requiring PLIF were randomly divided into two groups. All patients were intravenously injected with 1 g of TXA 15 min before skin resection. Every day after the surgery, 200 ml saline was intravenously injected for 1–3 days in Group A, while Group B received 1 g of TXA instead of saline. The total blood loss (TBL), IBL, PBL, HCT, Hb, blood transfusion volume, inflammation-related indicators, and complications were recorded.

**Results:**

TBL, PBL, and hidden blood loss (HBL) in Group B were significantly lower than those in Group A (*P* < 0.05). The maximum decreases in HCT and Hb in Group B were also significantly lower than those in Group A (*P* < 0.05), and the drainage removal time (DRT) was sooner in Group B than in Group A (*P* = 0.003). On the 3rd and 5th days after surgery, the level of CRP in Group B was significantly lower than that in Group A (*P* < 0.05). Similarly, IL-6 levels were significantly lower in Group B for the first 5 days postoperatively (*P* < 0.001). Sex, operation time, level of decompression, length of incision, and change in HCT were significant predictors of both TBL and HBL. TBL was also significantly associated with BMI and preoperative fibrinogen, while postoperative TXA was a significant predictor of HBL only.

**Conclusion:**

Intravenous injection of 1 g of TXA 15 min before skin resection combined with continuous intravenous injection of 1 g of TXA 1 to 3 days after PLIF can reduce postoperative bleeding and shorten the time to drainage tube removal. In addition, it can also inhibit the postoperative inflammatory response.

**Clinical trial registration:**

ChiCTR2200056210.

## Introduction

Hemorrhage is one of the greatest challenges in posterior lumbar interbody fusion (PLIF) surgery. Total blood loss (TBL) includes both intraoperative blood loss (IBL) and postoperative blood loss (PBL). Hidden blood loss (HBL) is mainly PBL. HBL accounts for 45 to 47% of TBL (1, 2) in spinal surgery. Massive HBL may increase the blood transfusion rate, infection rate, risk of complications, and adverse symptoms such as anemia, which seriously affect the speed of recovery after surgery, extend the length of hospital stay, increase medical costs, and even threaten patients' lives (3, 4). Therefore, how to reduce HBL effectively has become an urgent problem to be solved in PLIF surgery.

As an antifibrinolytic drug, TXA has been widely used in surgical hemostasis. TXA can strongly absorb the lysine-binding site of the fibrinogen (FIB) affinity site on plasminogen and fibrinolytic enzymes in a reversible competitive manner, which hinders the binding between fibrinolytic enzymes and fibrin, inhibits fibrin degradation caused by fibrinolytic enzymes (5), and reduces the formation of D-dimer, thereby achieving hemostasis. At the same time, TXA can modulate the immune response and reduce the postsurgical inflammatory response (6–8). Compared with topical administration after activation of the fibrinolytic system, preoperative intravenous administration of TXA can improve its speed and effectiveness by inhibiting thrombus decomposition (9). Concurrently, intravenous TXA has been shown to be effective in reducing IBL and transfusion requirements (5, 10, 11).

Although the optimal dose of TXA has not yet been determined, most previous studies have used one dose of 1 g of TXA before or during surgery to control wellbeing (9, 11–14), with no additional benefits associated with higher doses (12, 15). The half-life of TXA is 2–11 h, with most of the drugs expelled from the kidney within 24 h (16). However, Xie et al. (17) and Blani et al. (18) reported that fibrinolysis peaks at 6 h postoperatively and persisted for at least 24 h. Therefore, to inhibit fibrinolysis completely, a single dose of TXA is not sufficient. Since increasing the dose increases the risk of epilepsy (12, 19), another strategy that has been investigated and shown to effectively and safely reduce HBL in total knee and total hip arthroplasty is the administration of multiple doses (20, 21). There are only limited studies on multiple TXA use in PLIF. Therefore, this study aimed to investigate whether the strategy of multiple intravenous sequential use of TXA can better inhibit postoperative bleeding after PLIF compared with a single dose. After consideration of the half-life and the large number of studies reporting minimum Hb and HCT on the 3rd postoperative day (14, 22, 23), the effect of three additional doses of TXA administered at 24-h intervals was assessed.

## Materials and methods

### Sample size

The sample size was calculated using PASS 15 (NCSS, LLC, Kaysville, USA) statistical software. According to the result of our preliminary study, the HBL of Group A was approximately 552 ml and that of Group B was approximately 457 ml. In order to show an HBL difference with a power of 0.80 at a 95% significance level, we needed 57 patients for each treatment group. To compensate for the expected 5% dropout rate, we decided to include at least 60 patients in each group (24).

### Study design and patient population

This was a prospective, single-center, double-blind, randomized controlled clinical study. From February 2022 to June 2022, patients who underwent PLIF surgery were randomly divided into Group A and Group B. This study was approved by the Ethics Committee of the First Affiliated Hospital of Chongqing Medical University Review Board (2021-321) and registered on the Chinese Clinical Trials Registry (ChiCTR2200056210).

### Inclusion criteria

The inclusion criteria were as follows: (1) age between 18 and 80 years; (2) patients who underwent complete laminectomy PLIF treatment; and (3) written informed consent.

### Exclusion criteria

The exclusion criteria were as follows: (1) Hb: female < 110 g/L, male < 120 g/L; (2) patients with abnormal coagulation function, preoperative platelet count (PLT) < 100 × 10^9^/L, international normalized ratio (INR) > 1.4, prolonged activated partial thromboplastin time (APTT) > 1.4-fold normal; (3) patients who had taken antiplatelet aggregates, such as aspirin or anticoagulants, in the last month; (4) patients with a history of thromboembolisms; (5) presence of thrombosis on lower extremity deep venous ultrasonography; (6) allergic to TXA; (7) patients who are not recommended for surgery under general anesthesia after consultation with relevant experts due to serious heart or respiratory diseases and renal or liver dysfunction; (8) American Society of Anesthesiologists (ASA) physical status >III; (9) history of any psychiatric illness (e.g., depression, mania, and affective disorders.); (10) previous lumbar surgery; and (11) lumbar vertebral tumors, tuberculosis, infection, or lumbar vertebral fracture caused by trauma.

### Study design

All patients were randomly assigned into two groups by a sealed envelope method. The study had a double-blind design with products masked and coded so that the participants and treating clinicians were unaware of which product each patient was receiving. Patients in Group A were intravenously injected with 1 g of TXA (200 ml TXA sodium chloride injection) 15 min before skin resection, and 200 ml of normal saline was intravenously injected every day after surgery for 1–3 days (at 24-h intervals from the first infusion). Patients in Group B were intravenously injected with 1 g of TXA 15 min before skin resection and intravenously injected with 1 g of TXA every day after surgery for 1–3 days.

### Surgical technique

All operations were performed by a single surgeon with over 10 years of experience in spinal surgery. The patient was in a prone position under general anesthesia. After routine disinfection, the back fascia consistent with the midline skin incision was longitudinally divided, and the paravertebral muscles were dissected from the spinous process and lamina to expose the facet joints. The pedicle screws were implanted successively. After complete laminectomy and discectomy, autologous bone particles and intervertebral fusion cages were tilted into the intervertebral space. Then, the incision was rinsed, hemostasis was accomplished, two drainage tubes were placed, and layer-by-layer suturing was carried out to close the wound. Postoperative drainage of <50 mL in 24 h was considered standard for the removal of drainage tubes (25), and the removal time was recorded. The patient was discharged when the incision was no longer swelling or producing exudate after the drainage tube was removed.

### Outcome evaluation

The primary observational indicators of this study were HBL and PBL. The secondary observations include TBL, drainage removal time (DRT), length of hospitalization (LOS), interleukin-6 (IL-6) (the determination method was flow cytometry), C-reactive protein (CRP), a decline of hematocrit and hemoglobin level, TXA complications, liver and kidney function indicators, and coagulation function indicators.

TBL was estimated according to the formula of Nadler and Gross as follows: (1) preoperative blood volume (PBV) was calculated as follows: k_1_ × height (m)^3^ + k_2_ × weight (kg) + k_3_, where k_1_ = 0.3669, and k_2_ = 0.03219, k_3_ = 0.6041 for men and k_1_ = 0.3561, k_2_ = 0.03308, and k_3_ = 0.1833 for women (26). (2) TBL was calculated as follows: PBV × (HCT_pre_ – HCT_post_)/HCT_ave_, where HCT_pre_ is the preoperative hematocrit and HCT_post_ is the lowest postoperative HCT. By this time, the patient's fluid shifts would have been largely completed and hemodynamically stable. HCT_ave_ is the mean of HCT_pre_ and HCT_post_ (27). ΔHCT was calculated as follows: HCT_pre_ – HCT_post_.

Hidden blood loss = TBL – IBL – PBL, of which IBL was estimated by weighing the surgical sponges, measuring blood collected by the suction canisters, and subtracting all irrigation fluids added to the surgical field (1 g of blood = 1 ml of blood). Note: The wound exudates after the operation were ignored because of their small size and difficulty of measurement. If the drainage volume was <50 mL/24 h, extubation was performed. PBL = (WD_POD1_ × Hct_POD1_ + WD_POD2_ × Hct_POD2_)/HCT_ave_. WD_POD1_ = the volume of wound drainage on POD1; Hct_POD1_ = the Hct of wound drainage on POD1; and POD = postoperative days.

Hb, prothrombin time (PT), INR, APTT, and D-dimer (D-D) were measured before the operation and on the first, third, and 5th days after the operation. Venous Doppler ultrasonography examination was performed before the operation and 1 week and 1 month after surgery. If the patient had symptoms such as lower limb pain, swelling, and other symptoms, venous Doppler was performed. The incidence of thromboembolic events was tracked for 1 month after the operation. The maximum Hb drop was defined as the difference between the preoperative Hb and the minimal Hb level obtained postoperatively during hospitalization and before any blood transfusion.

An allogenic transfusion was given if the Hb level was <70 g/L or between 70 and 100 g/L with sustained blood loss or anemic symptoms (such as dizziness, palpitation, and paleness). The standard of transfusing fresh frozen plasma (FFP, 200 ml/time) was INR > 1.5 or APTT > 1.5-fold normal baseline, and the input standard of transfusing platelets (1 U/time) was PLT < 100 × 10^9^/L. If the patient received a blood transfusion, the post-transfusion Hb values were not included in the statistical analysis.

In addition, the general information collected for each patient included sex, age, body mass index (BMI), duration of operation, and hospitalization expenses.

### Statistical analysis

The Kolmogorov–Smirnov test was used to test whether the quantitative data obeyed the normal distribution in this study. The quantitative values in this study are expressed as the mean ± standard deviation or median (quartile), and the frequency and percentages of categorical data were calculated. The Mann–Whitney U-test and *t*-test were used for comparisons of the variables between the two groups. A multiple regression analysis was used to examine the variables that affected TBL and HBL. In cases with a *p*-value of < 0.05, the results were considered statistically significant. The statistical analysis was performed using SPSS 25.0 software (SPSS Inc, Chicago, Illinois).

## Results

### Patient information

**Figure 1:**
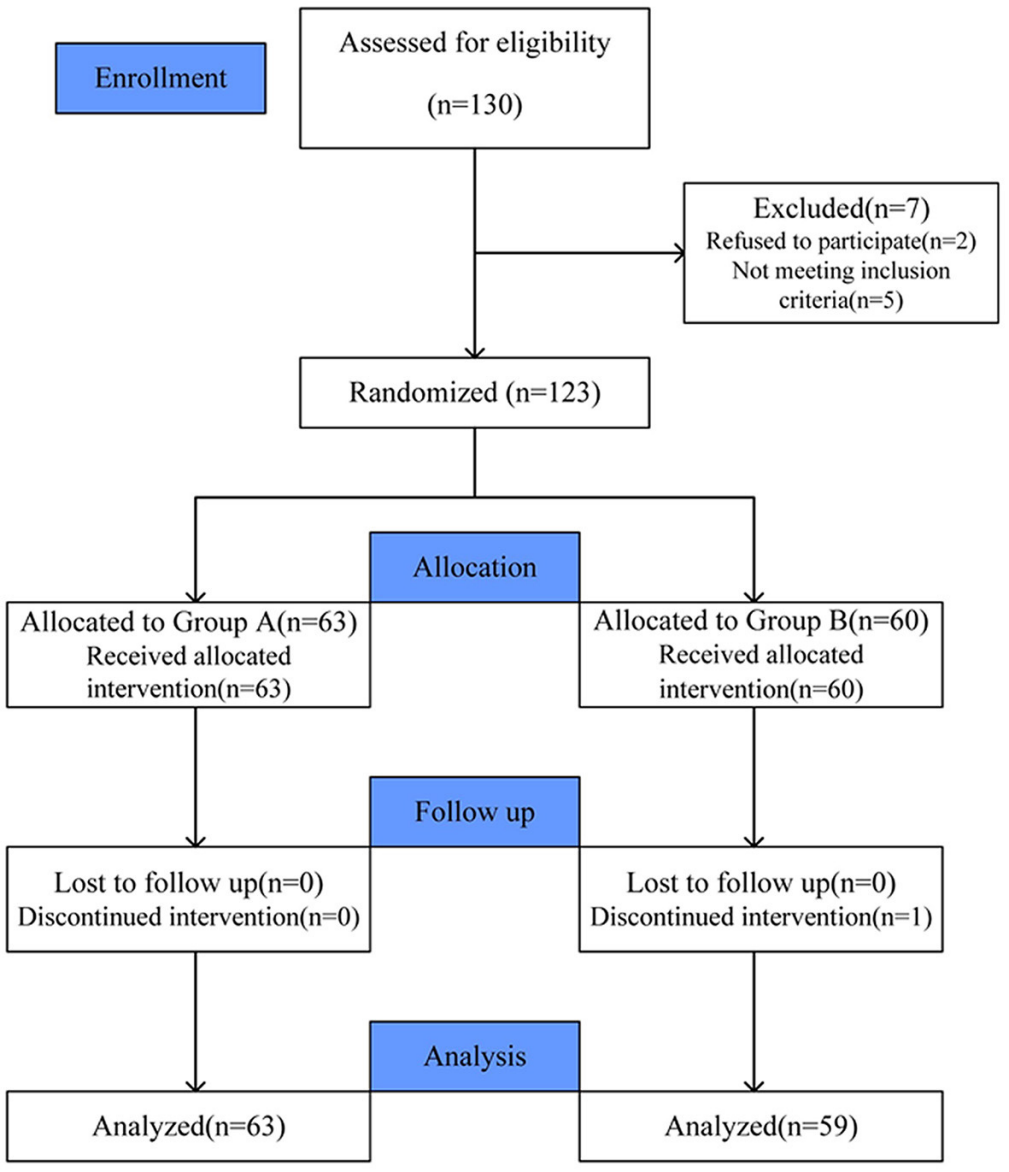
Patient recruitment and analysis.

Of 130 eligible consecutive patients, two patients refused to participate, and five patients did not meet the inclusion criteria. Then, 123 patients were enrolled and divided into Group A (63 cases) and Group B (60 cases). During the trial, one patient in Group B developed unbearable vomiting and palpitations after intravenous TXA and was discontinued. Finally, 122 patients were included in the final analysis (Figure 1). All patients underwent surgery successfully. There was no significant difference between the groups in demographic characteristics as shown in Table 1. There was also no significant difference in operation time, level of decompression, or length of incision (Table 2).

**Table 1:** Baseline demographic and clinical characteristics.

	**Group A (*n =* 63)**	**Group B (*n =* 59)**	**t/z**	***p*-value**
Age (years)	60.51 ± 9.30	58.66 ± 6.87	1.253	0.213
Sex (M/F)	26/37	24/35	0.066	0.947
Weight (kg)	63.93 ± 9.84	62.29 ± 9.35	0.943	0.348
Height (m)	1.60 ± 0.08	1.60 ± 0.08	0.283	0.777
BMI (kg/m^2^)	24.90 ± 3.26	24.36 ± 2.89	0.959	0.339
ASA grade (I/II/III)	1/41/21	1/40/18	0.331	0.741
Hypertension (*n*, %)	24/63 (38.1%)	13/59 (22.0%)	1.921	0.055
Smoke (*n*, %)	16/63 (25.4%)	13/59 (22.0%)	0.434	0.664
PBV (ml)	3,943 ± 624.8	3,873 ± 589.5	0.641	0.523

BMI, body mass index; ASA, American Society of Anesthesiologists; PBV, patient blood volume. Data are expressed as the mean ± SD or frequency (%).

**Table 2:** Intraoperative and postoperative parameters.

	**Group A (*n =* 63)**	**Group B (*n =* 59)**	**t/z**	***p*-value**
Operation time (min)	232.30 ± 53.21	224.46 ± 50.71	0.832	0.407
Level of decompression (1/2/3)	26/30/7	27/25/7	0.372	0.710
Length of incision (cm)	9.91 ± 1.86	9.41 ± 1.50	1.654	0.101
Blood transfusion (*n*, %)	2/63 (3.2%)	3/59 (5.1%)	0.530	0.596
DRT (d)	3.13 ± 0.42	2.92 ± 0.34	3.055	0.003^*^
MCVT (*n*, %)	1/63 (1.6%)	1/59 (1.7%)	0.047	0.963
LOS (days)	6.27 ± 0.52	6.07 ± 0.52	2.154	0.033^*^
Medical expenses (¥)	18,205.60 ± 1,935.38	17,983.54 ± 1,867.53	0.644	0.521

DRT, drainage tube removal time; MCVT, muscular calf vein thrombosis; LOS, length of hospitalization. Data are expressed as the mean±SD or frequency (%). ^*^indicates statistically significant differences (*P* < 0.05).

### Blood loss and blood transfusion-related parameters

**Figure 2:**
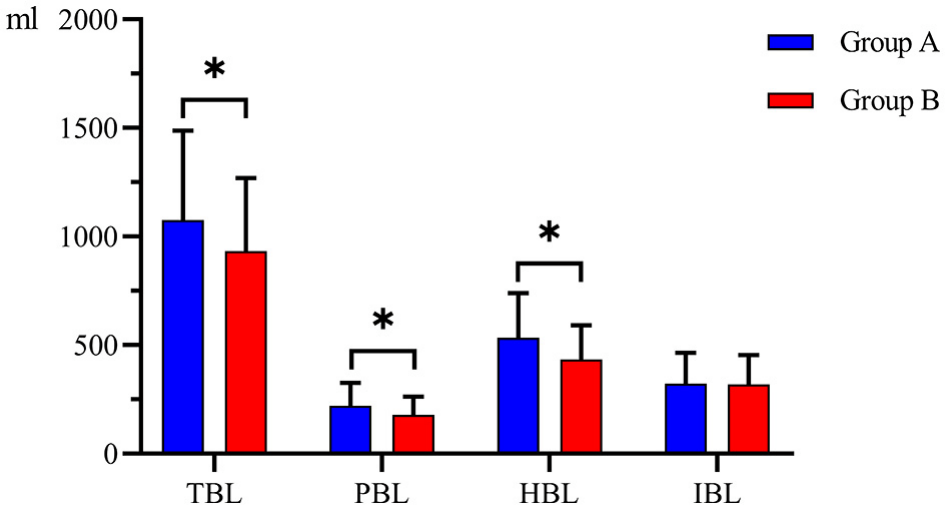
Blood loss between the two groups. Group A was preoperative single-dose intravenous TXA. Group B was sequential multi-dose intravenous TXA. TBL indicates total blood loss. IBL indicates intraoperative blood loss. PBL indicates postoperative blood loss. HBL indicates hidden blood loss. *indicates statistically significant differences (*P* < 0.05).

**Figure 3:**
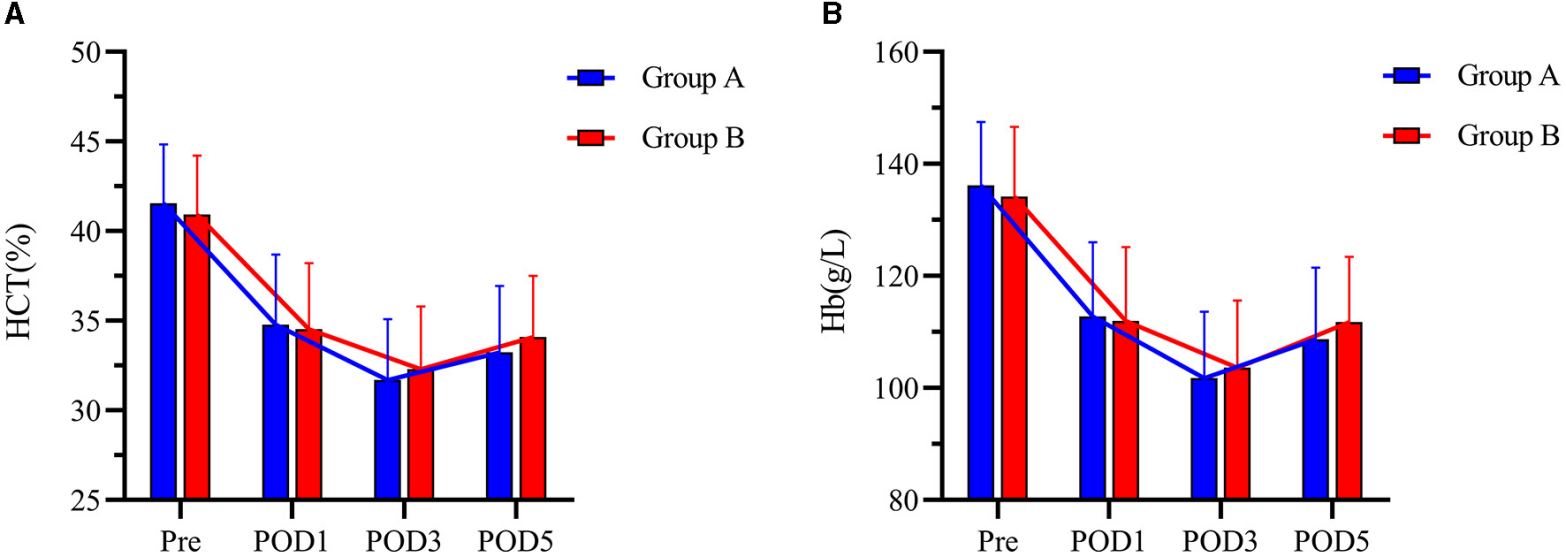
**(A, B)** HCT and Hb levels at Pre, POD1, POD3, and POD5 between the two groups. Pre represents preoperative. POD1 represents postoperative day 1. POD3 represents postoperative day 3. POD5 represents postoperative day 5. Group A was preoperative single-dose intravenous TXA. Group B was sequential multi-dose intravenous TXA.

There was no significant difference in IBL between the two groups (304.00 (227.25–360.25) ml and 287.00 (231.50–366.50) ml, *P* = 0.924). The mean TBL, PBL, and HBL in Group B (933.08 ± 335.80 ml, 178.56 ± 83.34 ml, 434.60 ± 155.87 ml) were significantly lower than those in Group A (1,076.49 ± 410.97 ml, mean difference = 143.41 ml, 95% CI, 8.36–278.47 ml, *P* = 0.038); (220.71 ± 104.83 ml, mean difference = 42.15 ml, 95% CI, 8.05–76.25 ml, *P* = 0.016); and (533.73 ± 205.44 mL, mean difference = 99.13 mL, 95% CI, 33.43–164.83 mL, *P* = 0.003) (Figure 2). There was no significant difference in HCT or Hb between the two groups preoperatively and postoperatively (Figure 3). It is interesting that the mean ΔHCT and ΔHb in Group B (8.63 ± 2.75%, 30.54 ± 9.66 g/L) were significantly lower than those in Group A (9.85 ± 3.29%, mean difference = 1.22%, 95% CI, 0.13–2.31%, *P* = 0.028; 34.44 ± 11.02 g/L, mean difference = 3.90 g/L, 95% CI, 0.18–7.63 g/L, *P* = 0.040) (Table 3). There was no significant difference in the blood transfusion rate between the two groups. There were five cases of intraoperative blood transfusion, including three cases in Group B and two cases in Group A (Table 2).

**Table 3:** Routine blood and coagulation parameters.

	**Group A (*n =* 63)**	**Group B (*n =* 59)**	**t/z**	***p-*value**
ΔHCT	9.85 ± 3.29	8.63 ± 2.75	2.218	0.028^*^
ΔHb	34.44 ± 11.02	30.54 ± 9.66	2.074	0.040^*^
FIB_Pre_	3.03 ± 0.56	2.76 ± 0.46	2.878	0.005^*^
FIB_POD1_	2.94 ± 0.58	2.97 ± 0.52	0.295	0.769
FIB_POD3_	3.95 ± 0.91	3.74 ± 0.69	1.393	0.166
FIB_POD5_	3.99 ± 1.03	3.74 ± 0.67	1.582	0.117
ΔFIB	0.92 ± 0.94	0.98 ± 0.71	0.415	0.679
FDP_Pre_	0.70 (0.40–1.13)	0.60 (0.40–0.95)	1.097	0.272
FDP_POD1_	4.10 (2.38–6.70)	2.40 (1.80–4.15)	2.619	0.009^*^
FDP_POD3_	3.95 (2.30–6.75)	2.80 (1.80–4.70)	2.726	0.006^*^
FDP_POD5_	5.90 (2.85–10.13)	4.60 (3.40–6.90)	1.002	0.316
D-D_Pre_	0.24 (0.16–0.41)	0.26 (0.14–0.38)	0.267	0.790
D-D_POD1_	1.56 (0.86–2.53)	1.04 (0.70–1.46)	2.400	0.016^*^
D-D_POD3_	1.17 (0.75–2.19)	0.84 (0.61–1.38)	2.393	0.017^*^
D-D_POD5_	2.32 (1.11–4.06)	1.91 (1.31–2.95)	0.679	0.497
IL-6_Pre_	3.04 ± 0.57	3.08 ± 0.60	0.378	0.706
IL-6_POD1_	98.91 ± 18.19	61.56 ± 11.97	13.479	< 0.001^*^
IL-6_POD3_	45.72 ± 8.42	32.98 ± 6.89	9.112	< 0.001^*^
IL-6_POD5_	25.32 ± 4.04	20.30 ± 3.45	7.364	< 0.001^*^
CRP_Pre_	2.51 (1.63–3.65)	2.27 (1.77–3.09)	0.433	0.665
CRP_POD1_	11.00 (7.06–15.60)	8.92 (5.88–17.60)	0.502	0.616
CRP_POD3_	16.25 (7.90–31.25)	12.30 (6.70–15.60)	2.211	0.027^*^
CRP_POD5_	6.99 (4.37–11.30)	5.10 (3.95–7.50)	2.075	0.038^*^

Pre, Preoperative; POD, postoperative days; HCT, hematocrit; Hb, hemoglobin; FIB, fibrinogen; FDP, fibrinogen degradation products; D-D, D-dimer; IL-6, Interleukin-6; CRP, C-Reactive Protein; ΔHCT, HCT_Pre_-HCT_post_; ΔHb, Hb_Pre_-Hb_post_; ΔFIB, FIB_post_-FIB_Pre_, where FIB_post_ is the highest postoperative FIB. Data are expressed as the mean ± SD or median (quartile). ^*^indicates statistically significant differences (*P* < 0.05).

A multiple regression analysis showed that TBL was positively correlated with BMI, operation time, level of decompression, length of incision, and ΔHCT. It was negatively correlated with preoperative levels of FIB (Table 4). HBL was positively correlated with operation time, length of incision, and ΔHCT. It was negatively correlated with the level of decompression and postoperative TXA (Table 5). As compared to male patients, female patients had a reduced risk of both TBL and HBL (Tables 4, 5).

**Table 4:** Multiple regression analysis for the influence of predictors on TBL.

	**Unstandardized coefficient**		**Standardized coefficient**		
**Predictors**	**B**	**SE**	**Beta**	**T value**	***p*** **-value**
Postoperative TXA administration	−30.703	21.646	−0.040	−1.418	0.159
Age (years)	1.362	1.247	0.029	1.092	0.277
Sex	−121.826	27.448	−0.158	−4.438	< 0.001^*^
BMI (kg/m^2^)	9.489	3.715	0.077	2.554	0.012^*^
Operation time (min)	0.742	0.283	0.101	2.624	0.010^*^
Level of decompression	29.668	13.664	0.104	2.171	0.032^*^
Smoke	−23.351	30.171	−0.026	−0.774	0.441
Length of incision (cm)	40.319	14.410	0.180	2.798	0.006^*^
ΔHCT	76.328	6.423	0.618	11.883	< 0.001^*^
FIB_Pre_	−61.955	22.555	−0.086	−2.747	0.007^*^

Adjust R^2^ = 0.921, *P* < 0.001. ^*^indicates statistically significant differences (*P* < 0.05).

**Table 5:** Multiple regression analysis for the influence of predictors on HBL.

	**Unstandardized coefficient**		**Standardized coefficient**		
**Predictors**	**B**	**SE**	**Beta**	**T value**	***p-*** **value**
Postoperative TXA administration	−30.066	11.463	−0.080	−2.623	0.010^*^
Age (years)	−0.582	0.660	−0.025	−0.882	0.380
Sex	−58.952	14.536	−0.154	−4.056	< 0.001^*^
BMI (kg/m^2^)	2.642	1.967	0.043	1.343	0.182
Operation time (min)	0.349	0.150	0.096	2.327	0.022^*^
Level of decompression	−45.606	7.236	−0.324	−6.303	< 0.001^*^
Smoke	−17.252	15.978	−0.039	−1.080	0.283
Length of incision (cm)	58.712	7.631	0.530	7.694	< 0.001^*^
ΔHCT	33.879	3.402	0.554	9.960	< 0.001^*^
FIB_Pre_	−5.867	11.945	−0.016	−0.491	0.624

Adjust R^2^ = 0.910, *P* < 0.001. ^*^indicates statistically significant differences (*P* < 0.05).

**Figure 4:**
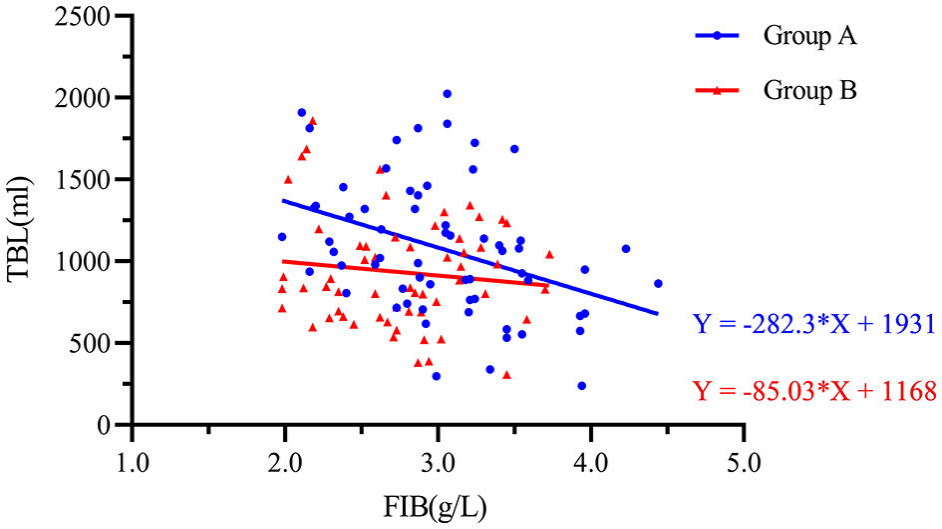
Scatter plot showing the negative logarithmic correlation between the preoperative fibrinogen concentration (g/L) and TBL (mL). Group A was preoperative single-dose intravenous TXA. Group B was sequential multi-dose intravenous TXA.

The preoperative FIB and TBL were analyzed by a scatter plot, and an equation was fitted. Figure 4 shows the scatter plot of the preoperative fibrinogen level and total bleeding volume. There was a negative correlation between these two variables. The fitted equation of Group A was Y = −282.3 × X + 1,931 and that of Group B was Y = −85.03 × X + 1,168. This result indicated that postoperative intravenous sequential TXA injection can attenuate the increase in TBL caused by low preoperative FIB.

### Inflammation markers

There was no significant difference in the IL-6 levels between the two groups preoperatively; however, IL-6 was significantly reduced in Group B compared to Group A 1–5 days postoperatively (*P* < 0.001). The CRP levels did not differ between the two groups at Pre and POD1. However, at 3 and 5 days after the operation, the CRP levels in Group B [12.30 (6.70–15.60) mg/L, 5.10 (3.95–7.50) mg/L] were lower than those in Group A [16.25 (7.90–31.25) mg/L, *P* = 0.027; 6.99 (4.37–11.30) mg/L, *P* = 0.038] (Table 3). This indicated that postoperative intravenous sequential application of TXA could reduce the inflammatory response.

### Coagulation indicators

**Figure 5:**
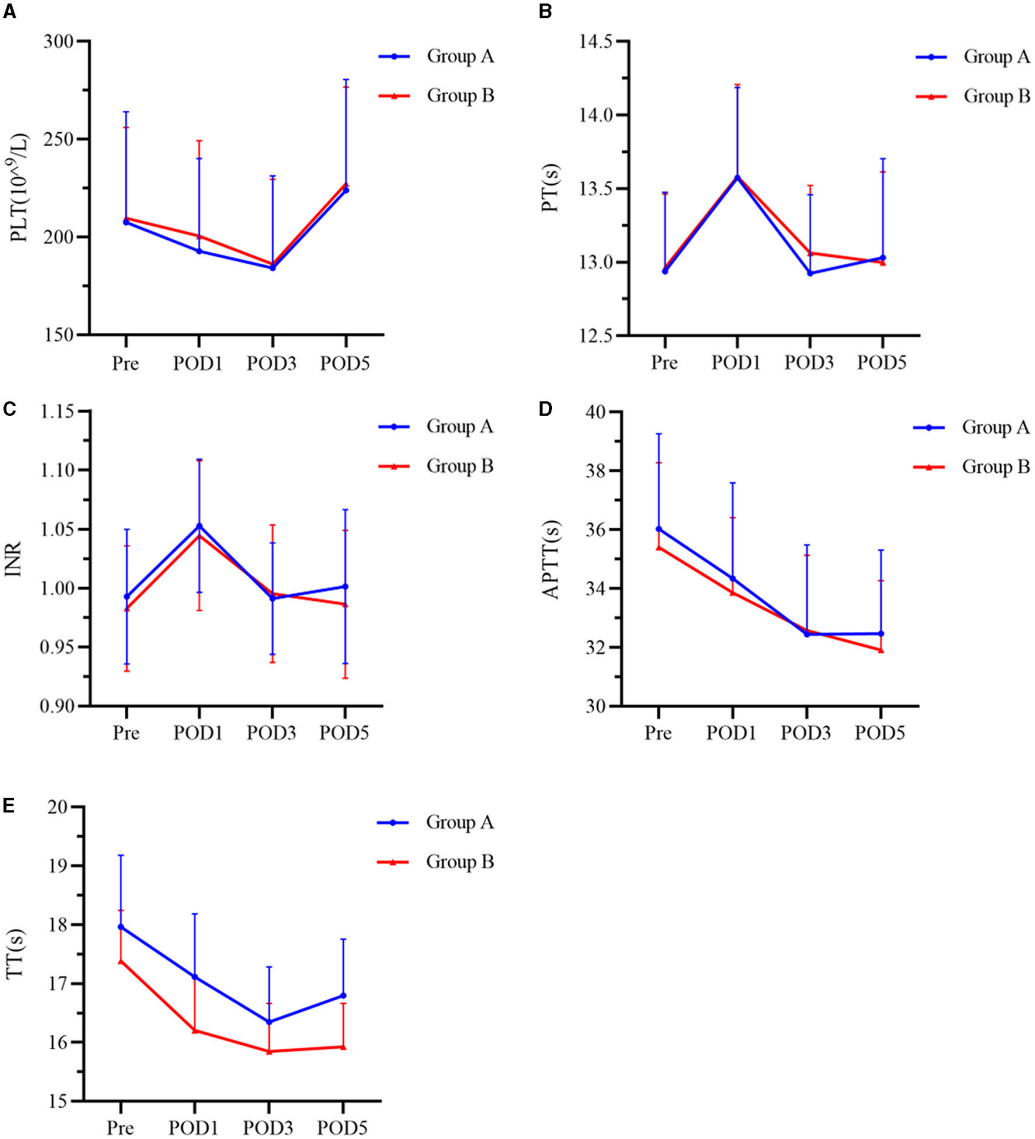
**(A–E)** Index of coagulation function at Pre, POD1, POD3, and POD5 between the two groups. Group A was preoperative single-dose intravenous TXA. Group B was sequential multi-dose intravenous TXA. PLT represents platelets, PT represents prothrombin time, INR represents international normalized ratio, APTT represents activated partial thromboplastin time, and TT represents thrombin time. Pre represents preoperative. POD1 represents postoperative day 1. POD3 represents postoperative day 3. POD5 represents postoperative day 5.

Coagulation parameters including PLT, PT, INR, APTT, and TT of all patients were changed after surgery but did not differ between the two treatment groups at any timepoint (Figure 5).

### Surgical outcomes and complications

In Group B, the DRT was earlier (2.92 ± 0.34 days vs. 3.13 ± 0.42 days, *P* = 0.003), and the LOS was shorter (6.07 ± 0.52 days vs. 6.27 ± 0.52 days, *P* = 0.033). One case of muscular calf vein thrombosis occurred in both groups (Table 2). No adverse reactions associated with blood transfusion and no drug-related complications, such as DVT, PEs, cardiac infarction, impaired hepatic and renal function, or epilepsy, were found in any patient (Table 6).

**Table 6:** Parameters of liver and kidney function.

	**Group A (*n =* 63)**	**Group B (*n =* 59)**	**t/z**	***p*-value**
Alb_Pre_	43.00 ± 2.86	42.11 ± 3.08	1.672	0.097
Alb_POD1_	38.00 ± 2.92	37.98 ± 2.81	0.052	0.959
Alb_POD3_	37.19 ± 2.60	36.27 ± 2.56	1.963	0.052
Alb_POD5_	38.15 ± 2.94	37.95 ± 2.99	0.381	0.704
ALT_Pre_	16.85 (13.00–25.03)	18.20 (14.40–28.10)	0.607	0.544
ALT_POD3_	18.00 (12.10–23.25)	18.00 (13.85–27.00)	0.415	0.678
AST_Pre_	17.00 (14.53–20.35)	17.70 (14.85–22.00)	0.923	0.356
AST_POD3_	15.00 (12.98–20.85)	17.50 (13.25–22.00)	1.545	0.122
BUN_Pre_	5.74 ± 1.48	6.26 ± 1.98	1.633	0.105
BUN_POD3_	6.48 ± 1.82	6.54 ± 1.64	0.211	0.833
SCr_Pre_	65.95 ± 14.52	67.08 ± 14.27	0.434	0.665
SCr_POD3_	60.05 ± 13.29	59.93 ± 13.03	0.048	0.961

Alb, albumin; ALT, alanine aminotransferase; AST, aspartate transaminase; BUN, blood urea nitrogen; SCr, serum creatinine. Data are expressed as the mean±SD or median (quartile).

## Discussion

Although blood conservation strategies, such as hypotensive anesthesia and avoiding abdominal compression, are employed, a spinal fusion operation may still lead to considerable blood loss. Therefore, safe and effective intervention strategies are still needed. In reviewing the scientific database of available literature on this topic, we found that TXA has been widely used in orthopedic surgery and has a positive hemostatic effect (10, 11, 28). At present, the optimal dose of TXA is not determined, however, a large number of studies use a TXA dose of 1 g (12, 15, 29, 30). The specification of TXA sodium chloride injection used in our study was 0.5 g/bottle. If considering weight adaption, it is difficult to achieve accurate dosage in clinical practice. Therefore, 1 g of TXA was selected, regardless of body weight.

The application of TXA in previous research mainly focused on preoperative or intraoperative assessments, but a few studies have focused on the role of HBL in PLIF and its regulatory factors (9, 31). Since Sehat et al. reported that HBL accounted for 49% of the total blood loss after total hip replacement, surgeons have gradually become aware that HBL plays an important role in perioperative TBL (32). This reveals why even if the patient's IBL or PBL seems acceptable, the patient may develop anemia after surgery, resulting in hypoperfusion coagulation dysfunction (33).

In our study, HBL accounted for 46.58% of TBL in Group B and 49.58% of TBL in Group A (Table 2). This result is slightly higher than that of Smorgick et al. and Xu et al. (1, 2). The multiple regression analysis showed that compared with women, male patients had a higher risk of increased TBL and HBL, which was consistent with the results of Cushner et al. (34). We speculate that this may be due to men having more muscle and less fat in their lower back than women (35) or because men have larger lumbar lamina (36), leading to more intraoperative and postoperative bleeding. The proportion of men in our study was 40.98% higher than that in Smorgick et al. (38.6%) and Xu et al. (30.0%) (1, 2). This may partially explain the slightly higher proportion of HBL in our study. At the same time, there were statistical differences between DRT and LOS in our study, but the mean difference between the two groups was not obvious and unlikely to be clinically significant. However, we further analyzed the data based on the level of decompression and found that there were no statistical differences in the number of DRT in Group A (2.96 ± 0.34) and Group B (2.93 ± 0.73, *P* = 0.822) on the level 1. On level 2, there were significant differences between Group A (3.13 ± 0.35) and Group B (2.96 ± 0.20, *P* = 0.025). On level 3, the number of DRT in Group A (3.71 ± 0.49) was significantly more than that in Group B (3.14 ± 0.38, *P* = 0.031). No difference in LOS between the two groups may be due to the small sample size and the single-center study design. Additional multi-center and larger samples are needed for evaluating whether multi-dose TXA can effectively shorten LOS. In this study, sequential perioperative intravenous TXA also did not reduce the rate of blood transfusion. This too may reflect the small number of patients in the study requiring blood transfusion (4.1%).

Many studies have confirmed the safety of using TXA with routine methods (37); however, the safety of postoperative intravenous sequential application of TXA still needs further investigation in consideration of an increased risk of DVT for higher doses of TXA or prolonged use (38–40). In our study, one patient in each group developed intermuscular venous thrombosis, but no other serious thromboembolism events occurred. There were no complications of DVT, PE, impaired hepatic or renal function, or myocardial infarction during the one-month follow-up. This may be because TXA does not increase the risk of thromboembolism (41) but is also likely due to the small sample size of the study, which is insufficient to find all adverse reactions.

The prethrombotic state, also known as the hypercoagulable state, refers to a pathological state characterized by an imbalance in coagulation and anticoagulant factors, which makes patients prone to thrombosis. Molecular marker levels (such as FIB, FDP, and D-D) are reliable indicators of the prethrombotic state (42). D-D is used as a marker for thrombosis and secondary fibrinolysis. Interestingly, the average value of D-D at POD3 in Group B [0.84 (0.61–1.38) mg/L] was significantly lower than that in Group A [1.17(0.75–2.19) mg/L, *P* = 0.017] (Table 3). This shows that the sequential application of TXA after the operation can effectively reduce the formation of D-D.

Fibrinolytic enzymes can induce monocytes to release proinflammatory cytokines and tissue factor expression, which can be inhibited by TXA in some instances (43). At the same time, TXA can also play an anti-inflammatory role by regulating a complement system (44). Relevant to this study, Wang et al. found that additional doses of TXA could further inhibit the postoperative inflammatory response (45). In the present study, we observed reduced proinflammatory IL-6 for 1–5 days post-operatively in patients treated with sequential TXA. Similarly, CRP levels were lower in this group on postoperative days 3 and 5, suggesting an anti-inflammatory effect associated with sequential multi-dose intravenous TXA, similar to the findings reported in previous studies (8, 45).

## Limitations

Our research has several limitations, which deserve further discussion. This was a single-center study, and the sample size was relatively small. Only the effect of 1 g of TXA was discussed in this study. Multi-center studies with larger numbers of patients and different doses are needed to confirm the beneficial effect of TXA to reduce postoperative and hidden blood loss after PILF.

## Conclusion

This study showed for the first time that sequential intravenous application of 1 g of TXA at 24-h intervals 1–3 days after the operation could effectively reduce TBL, PBL, and HBL without increasing complications. In addition, this treatment regime could also inhibit the postoperative inflammatory response and shorten the time to drainage tube removal. This study provides new ideas for bleeding management after spinal fusion.

## Data availability statement

The raw data supporting the conclusions of this article will be made available by the authors, without undue reservation.

## Ethics statement

The studies involving human participants were reviewed and approved by Ethics Committee of the First Affiliated Hospital of Chongqing Medical University Review Board (2021-321) and registered on Chinese Clinical Trials Registry (ChiCTR2200056210). The patients/participants provided their written informed consent to participate in this study.

## Author contributions

WJ and ZH worked on the design and setting up of the study. WD and YL performed the data analysis and drafted the manuscript. DL, ZM, and MC were responsible for collecting the data. XZ, JS, NZ, and JH performed the PLIF surgery. All authors have critically reviewed and approved the final manuscript.
